# Hydrogen Sulfide Inhibits Transforming Growth Factor-β1-Induced EMT via Wnt/Catenin Pathway

**DOI:** 10.1371/journal.pone.0147018

**Published:** 2016-01-13

**Authors:** Lin Guo, Wen Peng, Jie Tao, Zhen Lan, Hongya Hei, Lulu Tian, Wanma Pan, Li Wang, Xuemei Zhang

**Affiliations:** 1 Department of Pharmacology, School of Pharmacy, Fudan University, 826 Zhangheng Road, Pudong New District, Shanghai, 201203, China; 2 Department of Nephrology, Putuo Hospital, Shanghai University of Traditional Chinese Medicine, 164 Lanxi Road, Shanghai, 200062, PR China; Casey Eye Institute, UNITED STATES

## Abstract

Hydrogen sulfide (H_2_S) has anti-fibrotic potential in lung, kidney and other organs. The exogenous H_2_S is released from sodium hydrosulfide (NaHS) and can influence the renal fibrosis by blocking the differentiation of quiescent renal fibroblasts to myofibroblasts. But whether H_2_S affects renal epithelial-to-mesenchymal transition (EMT) and the underlying mechanisms remain unknown. Our study is aimed at investigating the *in vitro* effects of H_2_S on transforming growth factor-β1 (TGF-β1)-induced EMT in renal tubular epithelial cells (HK-2 cells) and the associated mechanisms. The induced EMT is assessed by Western blotting analysis on the expressions of α-SMA, E-cadherin and fibronectin. HK-2 cells were treated with NaHS before incubating with TGF-β1 to investigate its effect on EMT and the related molecular mechanism. Results demonstrated that NaHS decreased the expression of α-SMA and fibronectin, and increased the expression of E-cadherin. NaHS reduced the expression of TGF-β receptor type I (TβR I) and TGF-β receptor type II (TβR II). In addition, NaHS attenuated TGF-β1-induced increase of β-catenin expression and ERK phosphorylation. Moreover, it inhibited the TGF-β1-induced nuclear translocation of ββ-catenin. These effects of NaHS on fibronectin, E-cadherin and TβR I were abolished by the ERK inhibitor U0126 or β-catenin inhibitor XAV939, or β-catenin siRNA interference. We get the conclusion that NaHS attenuated TGF-β1-induced EMT in HK-2 cells through both ERK-dependent and β-catenin-dependent pathways.

## Introduction

Hydrogen sulfide (H_2_S) is an endogenous gaseous physiological molecule, produced in mammalian tissues from L-cysteine mainly by two pyridoxal-5’-phosphate-dependent enzymes, cystathionine β-synthetase (CBS) and cystathionine γ-lyase (CSE), and 3-mercaptopyruvate sulfurtransferase (3-MST) along with cysteine aminotransferase (CAT)[1–3]. Recent study by K. Jung et. al. has shown that in ureteral obstruction (UO)-induced kidney fibrosis, the levels of CBS and CSE, and the H_2_S concentration are decreased in kidney, whereas sodium hydrosulfide (NaHS, a H_2_S producer) diminished the suppressing effect of UO on CBS, CSE and H_2_S. In the meantime, treatment with NaHS also reduced the activation of the transforming growth factor-β1 (TGF-β1) signaling caused by UO, suggesting an inverse relationship between the H_2_S level and the in kidney fibrosis [4], opening the possibility for H_2_S as a potential therapeutical target for kidney fibrosis, and our study aimed to investigate the mechanism of anti-fibrotic effect of H_2_S.

The role of TGF-β signaling in kidney fibrosis is attributed to its ability to induce epithelial-to-mesenchymal transition (EMT)[5]. EMT is a kind of phenotypical change in epithelial cells at which circumstances they lose cell-cell basement membrane contacts and structural polarity [6]. As a result, the epithelial cells become spindle-shaped and morphologically similar to mesenchymal/myofibroblast cells [6]. The abnormal induction of EMT in kidney has been shown to contribute to tubulointerstitial fibrosis, the final common path to renal fibrosis [7].

TGF-β induces EMT via Smad-dependent and non-Smad signaling pathways [5]. Transcription factors identified downstream of TGF-β signaling include Snail/Slug, Twist, ZEB1 and ZEB2/Sip1, and Smads.

Apart from Smads pathway, there exists evidence supporting that TGF-β1 plays its role through ERK pathway. In several types of cells, such as pancreatic cancer cells and mammary epithelial cells, ERK is activated in TGF-β1-induced EMT and Erbin, a member of LAP family, inhibits TGF-β1-induced EMT by suppressing ERK activation [8]. β-catenin/TCF/LEF of the Wnt signaling pathway has also been shown to mediate EMT [9]. Wnt-independent β-catenin transactivation was observed due to loss of E-cadherin and consequent release of free β-catenin, mimicking Wnt signaling [10]. There is an apparent redundancy of the transcription factors involved in TGF-β1-mediated EMT, suggesting that none of these factors orchestrates EMT on its own. Rather, they may act synergistically when combined [11]. Furthermore, the observations that β-catenin nuclear translocation being Smads-dependent and that LEF/TCF signaling together with the reduction of TGF-β1-induced α-SMA expression in β-catenin null cells, strongly suggested an interaction between TGF-β1 and Wnt/β-catenin signaling [12, 13].

Given the effects of NaHS in the UO-induced kidney fibrosis, we hypothesized that NaHS may exerts its anti-fibrotic effect by influencing EMT process through both Smad-dependent and Smad-independent pathways like MAPK pathway and Wnt/catenin pathway, of which the latter is our interest and focus. In this study, we investigated the effect of NaHS on TGF-β1-induced EMT in human proximal tubular epithelial cells (HK-2 cells) and the underlying mechanisms related to ERK and Wnt/catenin pathways.

## Materials and Methods

### Reagents

NaHS (Sodium hydrosulfide) was purchased from Sigma (USA). To prepare stock solution, NaHS is dissolved in PBS (135 mM NaCl, 2.7 mM KCL, 1.5 mM KH_2_PO4, 8 mM Na_2_HPO4) to the concentration of 1M. Recombinant Human transforming growth factor beta 1 (TGF-β1) is purchased from R&D Systems (USA). The inhibitor U0126 is purchased from Cell Signaling Technology and reconstituted with DMSO to 10 mM stock solution according to the product instruction. The inhibitor XAV939 is purchased from Sigma and reconstituted with DMSO to 10 mM stock solution according to the product instruction.

The primary antibodies to phospho-ERK1/2, ERK1/2, phospho-Catenin and Non-phospho (Active) β-Catenin were purchased from Cell Signaling Technology (USA). The primary antibodies to α-SMA and E-cadherin were purchased from Epitomics (USA). The primary antibodies to TGF-β receptor I, CBS and GAPDH were purchased from Santa Cruz Biotechnology (CA, USA). Antibody to Fibronectin was purchased from Sigma (USA). The secondary AlexaFluor488-conjugated anti-rabbit antibody for Immunofluorescent staining was purchased from Invitrogen. BCA Protein Assay Kit was purchased from Shanghai Biocolor BioScience & Technology Company (Shanghai, China). β-catenin siRNA and control siRNA were purchased from Santa Cruz Biotechnology (CA, USA).

### Cell Lines and Cell Culture

Human proximal tubular epithelial cells (HK-2) were purchased from American Type Culture Collection (Rockville, MD, USA). HK-2 cells were cultured in RPMI 1640 containing 2000 mg/L NaHCO_3_, 10% FBS (GIBCO BRL, Co. Ltd, USA), 100 U/ml penicillin and 100 μg/ml streptomycin in a 5% CO_2_ incubator at 37°C. When reached 90% confluence, the cells were trypsinized and subcultured by a 1:3 split ratio in new culture flasks. For nuclear translocation experiment, cells were seeded at proper density on cell slides in 24-well plates.

### Western blot analysis

HK-2 cells were harvested, rinsed twice with ice-cold PBS and re-suspended in RIPA lysis buffer (150 mM NaCl, 50 mM Tris-HCl pH 7.4, 1 mM PSFM, 1 mM EDTA, 1% Triton X-100, 0.1% SDS). The liquid supernatant was collected after centrifugation at 10,000×g at 4°C for 15 min and the protein concentration was detected by BCA Protein Assay. After the addition of 5× Loading buffer, protein was boiled at 95°C for 10 min, which would be loaded and separated by equal amounts in 10% sodium dodecyl sulfate polyacrylamide gels (SDS-PAGE) and transferred onto PVDF membranes. The membranes were blocked with 5% milk in Tris-buffered saline with 0.1% Tween (TBST) at room temperature for 2 hours and then incubated with primary antibodies at 4°C overnight followed by incubated with horseradish peroxidase (HRP)-conjugated secondary antibody at room temperature for 2 hours. The enhanced chemiluminescent (ECL) substrate was used to visualize the protein blots and β-actin or GAPDH used as loading control.

### Immunofluorescent staining

HK-2 cells cultured on cell slides were fixed in 4% formaldehyde for 30 minutes, permeabilized in 0.1%Triton X-100 for 10 minutes and blocked in 5% bovine serum albumin (BSA) in PBS buffer for 1 hour at room temperature. The primary antibody for non-phosphorylated β-catenin (1:500, Cell Signaling Technology, USA) was incubated overnight at 4°C. The secondary AlexaFluor488-conjugated anti-rabbit antibody (Invitrogen, Carlsbad, CA) was used at a 1:150 dilution for 1 hour at RT. All antibodies were diluted with PBST containing 5% bovine serum albumin. Images were obtained using a Leica TCS SP5 confocal system confocal microscopy (Leica, Wetzlar, Germany)

### RNA interference

HK-2 cells were transfected with β-catenin siRNA or negative control siRNA using Lipofectamine 2000 (Invitrogen, Carlsbad, CA, USA) according to the manufacturer’s instruction.

### Cell migration

Comparative migration assay were conducted using a conventional 24-well Transwell plates of 6.5-mm diameter with polycarbonate membrane filters containing 8-μm pores (6.5 mm Transwell (#3422), Corning, NY, USA). Pretreatment of 100 uM NaHS is given to HK-2 cells for 12 hours and then TGF-β1 was given to the cells for 36 hours. Then, HK-2 cells were detached with trypsin-EDTA and resuspended in media without FBS to obtain cell suspension of 2×10^5^/ml. 500 μL growth medium (1640 with 20% FBS) was added to the lower wells of the chambers. A volume of 100 μL containing 2×10^4^ cells was seeded to the upper section of the chamber, and then incubated for 24 h in a 5% CO_2_ incubator at 37°C. The non-migrated cells were removed from the upper side of the membranes by cotton swabs. Cells that had gone through the membrane were fixed in 4% formaldehyde for 20 minutes and stained with 0.1% crystal violet solution. Three randomly microscope fields in each slide were chosen to calculate the invaded cells.

### Statistical analysis

All data were shown as mean ±SEM. Statistical differences were obtained by one-way ANOVA followed by Tukey’s multiple comparison test. Differences with values of p<0.05 were considered significant.

## Results

### 1. NaHS attenuated TGF-β1-stimulated EMT in HK-2 cells

**Fig 1:**
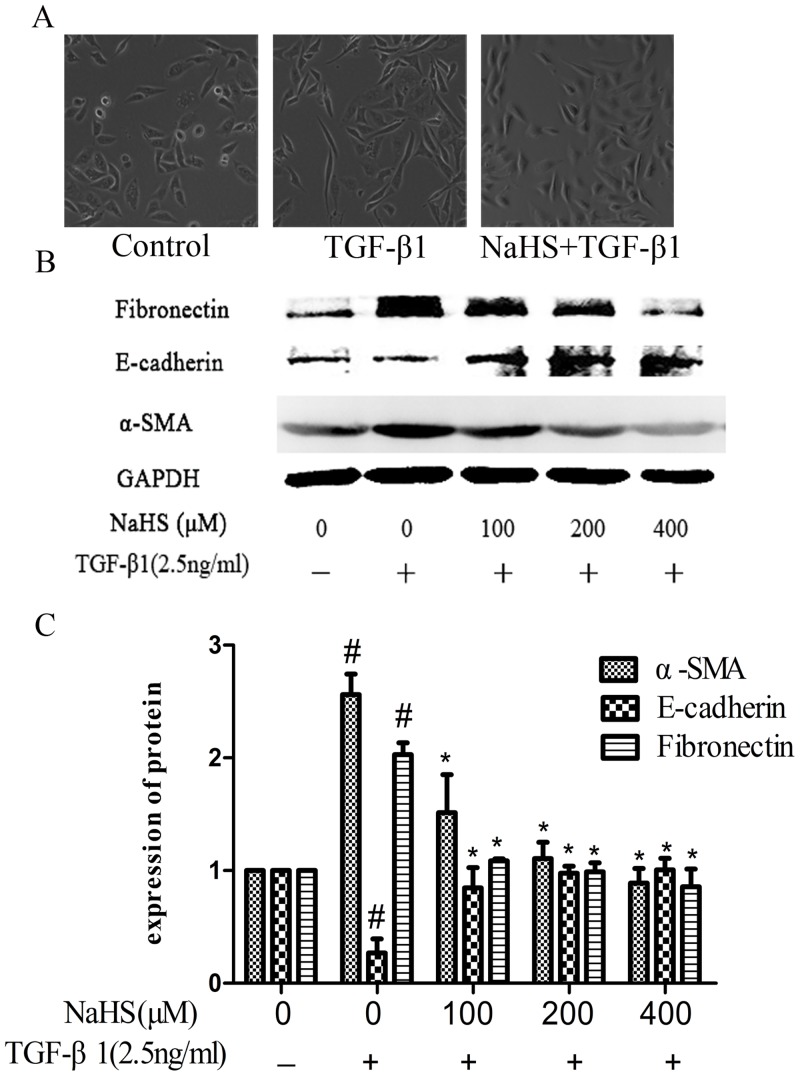
NaHS attenuated TGF-β1-stimulated EMT in HK-2 cells. Pretreatment by the several concentrations of NaHS is given to HK-2 cells for 12 hours and then TGF-β1 was given to the cells for the following 36 hours. (A) The influence of 400μM NaHS on the morphological change of HK-2 cells (B) Western blot assay for fibronectin, E-cadherin and α-SMA expressions in HK-2 cells. (C) Graphical representation of the relative quantification for fibronectin, E-cadherin and α-SMA. The relative values were calculated by the density of fibronectin, E-cadherin and α-SMA vs GAPDH (%). The values of mean ± SEM (n = 3) were gained from relative abundance quantified by densitometry and normalized to GAPDH. ^#^P<0.05 vs. control group, *P<0.05 vs TGF-β1 group. One-way ANOVA followed by Tukey’s multiple comparison test.

As shown in Fig 1, the HK-2 cell morphology has changed from normal to spindle-shape with TGF-β1 treatment compared to that of the untreated cells, and this change was attenuated by pretreatment of NaHS (Fig 1A). These changes are accompanied by the increase of the expression of α-SMA and fibronectin, and the decrease of the expression of E-cadherin in the TGF-β1 treated cells (Fig 1B & 1C), whereas NaHS pretreatment could down-regulate both α-SMA and fibronectin and could up-regulate E-cadherin (Fig 1B & 1C).

### 2. NaHS increased the expression of Cystathionine β-synthase (CBS)

**Fig 2:**
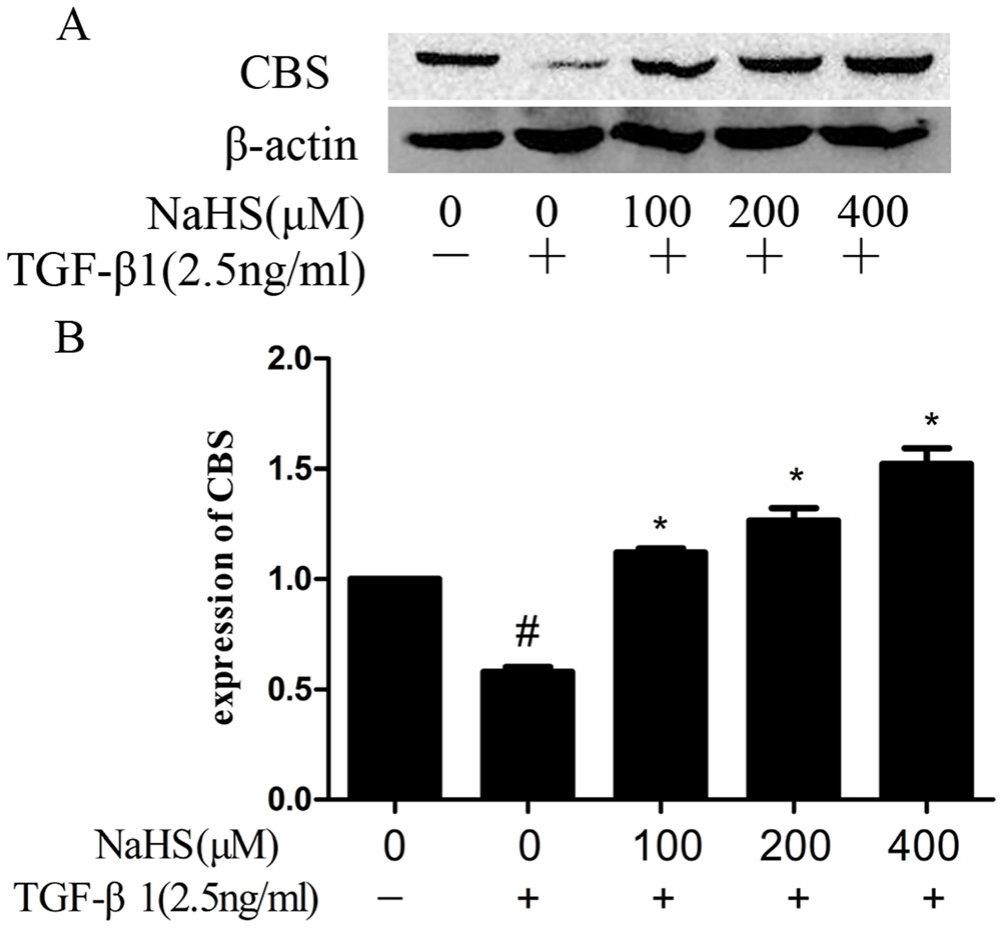
NaHS increased the Cystathionine β-synthase (CBS) expression in HK-2 cells. Pretreatment by the several concentrations of NaHS is given to HK-2 cells for 12 hours and then TGF-β1 was given to the cells for the following 36 hours. (A) NaHS increased the CBS expression level in a concentration‑dependent manner in TGF-β1-stimulated HK-2 cells. (B) Graphical representation of the relative quantification for CBS. The relative values were calculated by the density of of CBS vs β-actin (%). The values of mean ± SEM (n = 3) were gained from relative abundance quantified by densitometry and normalized to β-actin. ^#^P<0.05 vs. control group, *P<0.05 vs TGF-β1 group. One-way ANOVA followed by Tukey’s multiple comparison test.

Cystathionine γ-lyase (CSE) and CBS generate H_2_S [2]. We studied whether these enzymes are the mediators of the effect of NaHS in EMT. As shown in Fig 2, the expression of CBS is decreased significantly in TGF-β1 treated HK-2 cells. NaHS up-regulated CBS. However, we failed to detect the expression of CSE.

### 3. NaHS decreased the TGF-β1-induced overexpression of TGF-β receptor type II (TβR II) and TGF-β receptor type I (TβR I)

**Fig 3:**
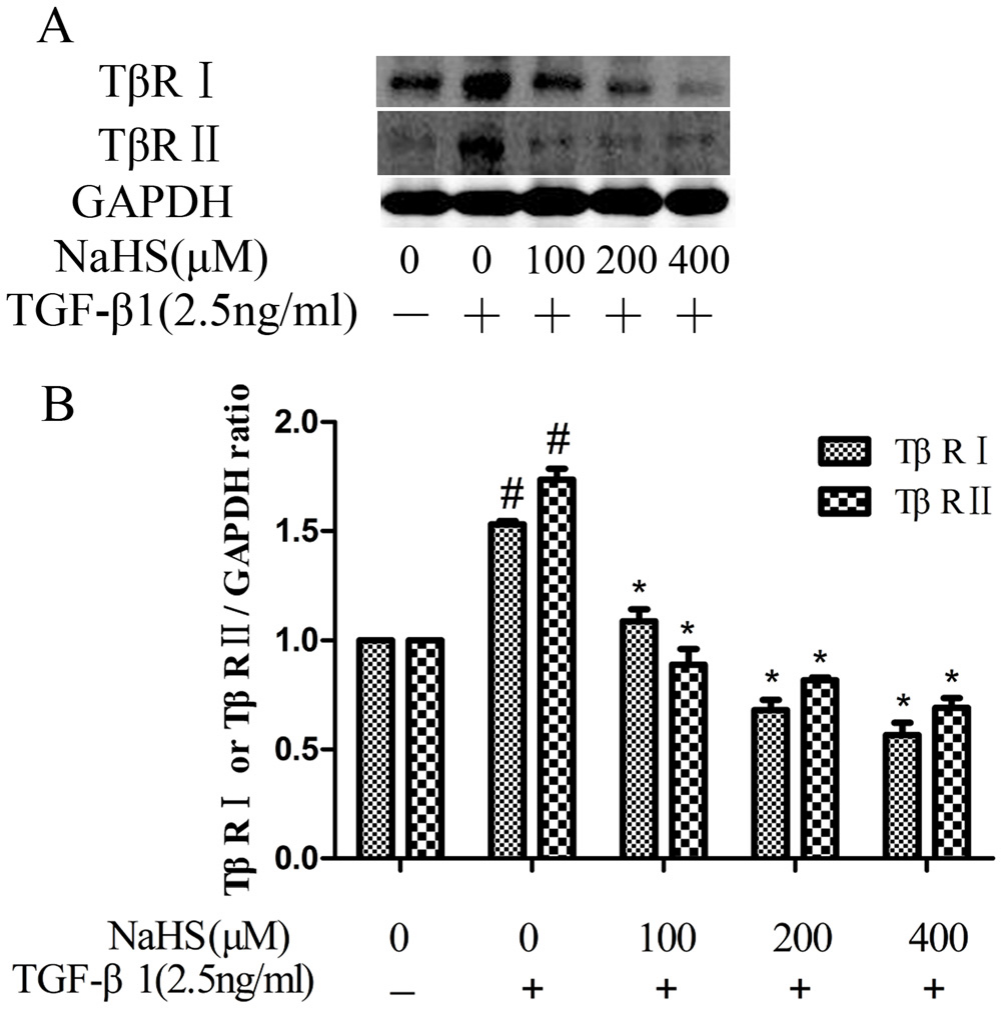
NaHS decreased the TGF-β1-stimulated overexpression of TGF-β receptor type II (TβR II) and TGF-β receptor type I (TβR I). Pretreatment by the several concentrations of NaHS is given to HK-2 cells for 12 hours and then TGF-β1 was given to the cells for the following 36 hours. (A) NaHS decreased both TβR I and TβR II overexpression stimulated by TGF-β1 in a concentration-dependent manner in HK-2 cells. (B) Graphical representation of the relative quantification for TβR I and TβR II. The relative values were calculated by the density of TβR I and TβR II vs GAPDH (%). The values of mean ± SEM (n = 3) were gained from relative abundance quantified by densitometry and normalized to GAPDH. ^#^P<0.05 vs. control group, *P<0.05 vs TGF-β1 group. One-way ANOVA followed by Tukey’s multiple comparison test.

TGF-β signaling cascade starts with TGF-β1 binding to TGF-β receptor type II (TβR II) and then the complex phosphorylates TGF-β receptor type I (TβR I)[5]. We studied effect of NaHS on TβR II and TβR I. As shown in Fig 3, NaHS lowered both the TβR II and TβR I expression levels in renal tubular epithelial cells.

### 4. NaHS decreased TGF-β1-induced overexpression of phosphorylated ERK1/2

**Fig 4:**
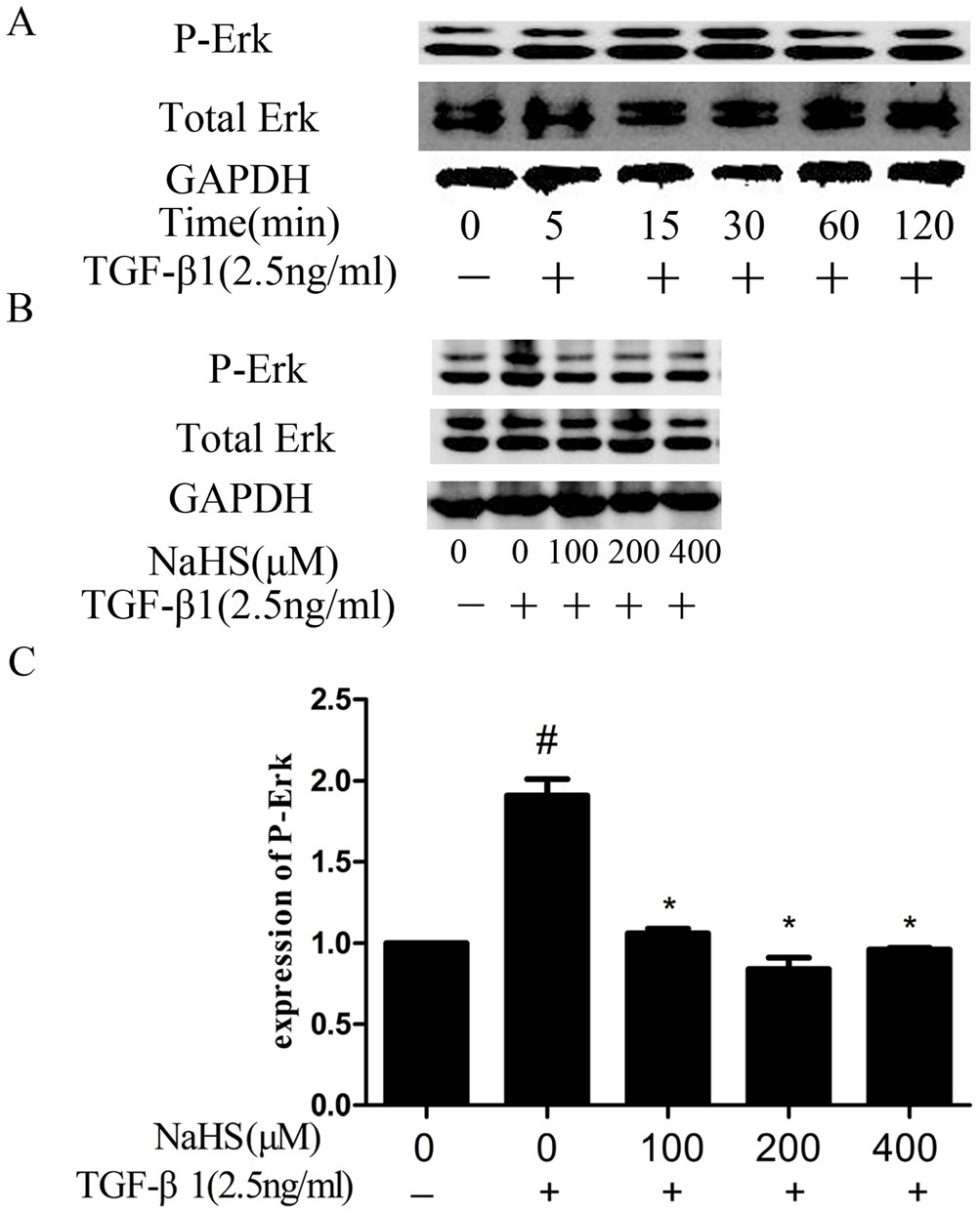
NaHS decreased TGF-β1-stimulated overexpression of phosphorylated ERK1/2. (A) HK-2 cells were incubated with TGF-β1 for several specific time periods, and the phosphorylated ERK1/2 was detected by western blot assay. (B) Western blot assay for phospho-ERK1/2 expression in HK-2 cells. Pretreatment by the several concentrations of NaHS is given to HK-2 cells for 12 hours and then TGF-β1 was given to the cells for 30 minutes at which time the phosphorylated ERK1/2 express the most (C) Graphical representation of the relative quantification for phospho-ERK1/2. The relative values were calculated by the density of phospho-ERK1/2 vs GAPDH (%). The values of mean ± SEM (n = 3) were gained from relative abundance quantified by densitometry and normalized to GAPDH. ^#^P<0.05 vs. control group, *P<0.05 vs TGF-β1 group. One-way ANOVA followed by Tukey’s multiple comparison test.

TGF-β can also activate ERK1/2 [14], thus we studied whether NaHS affect the activation level of ERK1/2. As shown in Fig 4, the ERK1/2 was activated by TGF-β1 and the phosphorylation level reach peak at 30 minutes (Fig 4A). HK-2 cells were pretreated by NaHS for 12 hours and then treated by TGF-β1 for half an hour. This significantly attenuated the ERK activation (Fig 4B & 4C). Results suggested that NaHS may inhibit TGFβ1-induced EMT via ERK-dependent signal pathway.

### 5. NaHS decreased TGF-β1-induced overexpression of non-phosphorylated β-catenin

**Fig 5:**
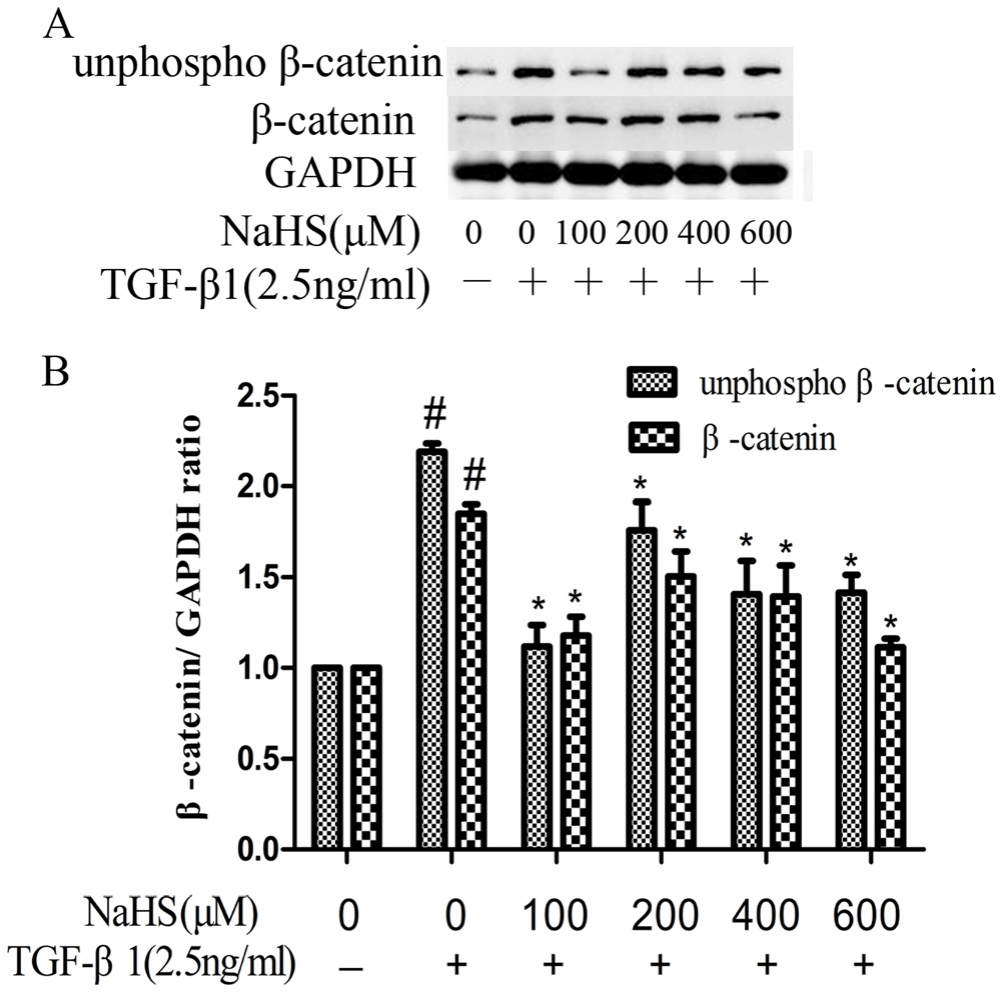
NaHS decreased TGF-β1-stimulated overexpression of non-phosphorylated β-catenin. HK-2 cells were pretreated with NaHS at the indicated doses for 12 hours and then they were treated by TGF-β1 for the following 36 hours. (A) Western blot assay for β-catenin and non-phosphorylated β-catenin expression in HK-2 cells. (B) Graphical representation of the relative quantification for β-catenin and non-phosphorylated β-catenin. The relative values were calculated by the density of β-catenin and non-phosphorylated β-catenin vs GAPDH (%). The values of mean ± SEM (n = 3) were gained from relative abundance quantified by densitometry and normalized to GAPDH. ^#^P<0.05 vs. control group, *P<0.05 vs TGF-β1 group. One-way ANOVA followed by Tukey’s multiple comparison test.

Under normal conditions, β-catenin is phosphorylated at its GSK3β sites and degraded. Upon stimulus, this GSK3β mediated phosphorylation is inhibited and non-phosphorylated β-catenin can translocate to the nucleus. Both total β-catenin and non-phosphorylated β-catenin expression levels were increased in HK-2 cells after TGF-β1 treatment (Fig 5). Pretreatment with NaHS for 12 hours decreased both non-phosphorylated β-catenin and β-catenin expression levels. NaHS at the concentration of 100 μM showed the best suppressing effect towards non-phosphorylated β-catenin (Fig 5).

### 6. NaHS inhibited nuclear translocation of non-phosphorylated β-catenin

**Fig 6:**
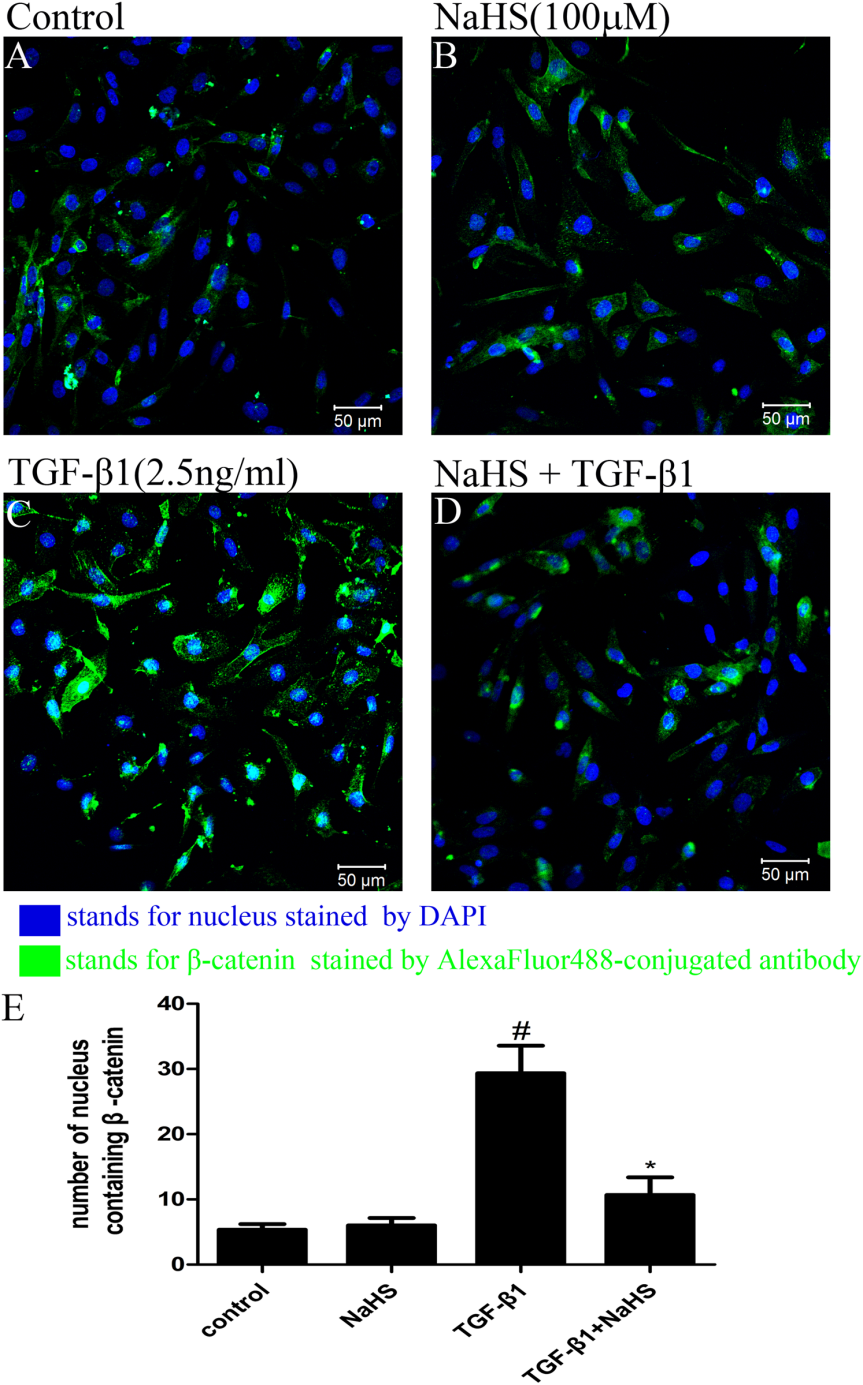
NaHS inhibited the nuclear translocation of non-phosphorylated β-catenin. (A,B,C,D) HK-2 cells were pretreated with or without NaHS (100μM) for 12 hours and then they were treated by TGF-β1 for the following 36 hours. Blue color stands for nucleus stained by DAPI and green color stands for non-phosphorylated β-catenin. (E) Graphical representation of the cells in which non-phosphorylated β-catenin translocated into nucleus. The values of mean ± SEM (n = 3) were gained from three randomly microscope fields in each slide. ^#^P<0.05 vs. control group, *P<0.05 vs TGF-β1 group. One-way ANOVA followed by Tukey’s multiple comparison test.

β-catenin is a nuclear transcription factor and a key element in Wnt/catenin pathway. The nuclear translocation of β-catenin is crucial for mediating EMT [15]. As shown in Fig 6, TGF-β1 promoted the nuclear translocation of non-phosphorylated β-catenin and pretreatment with NaHS diminish this effect, indicating that NaHS may suppresse EMT by inhibiting the nuclear translocation of non-phosphorylated β-catenin.

### 7. The anti-EMT effect of NaHS on HK-2 cells was blocked by the inhibitors of ERK1/2 and β-catenin

**Fig 7:**
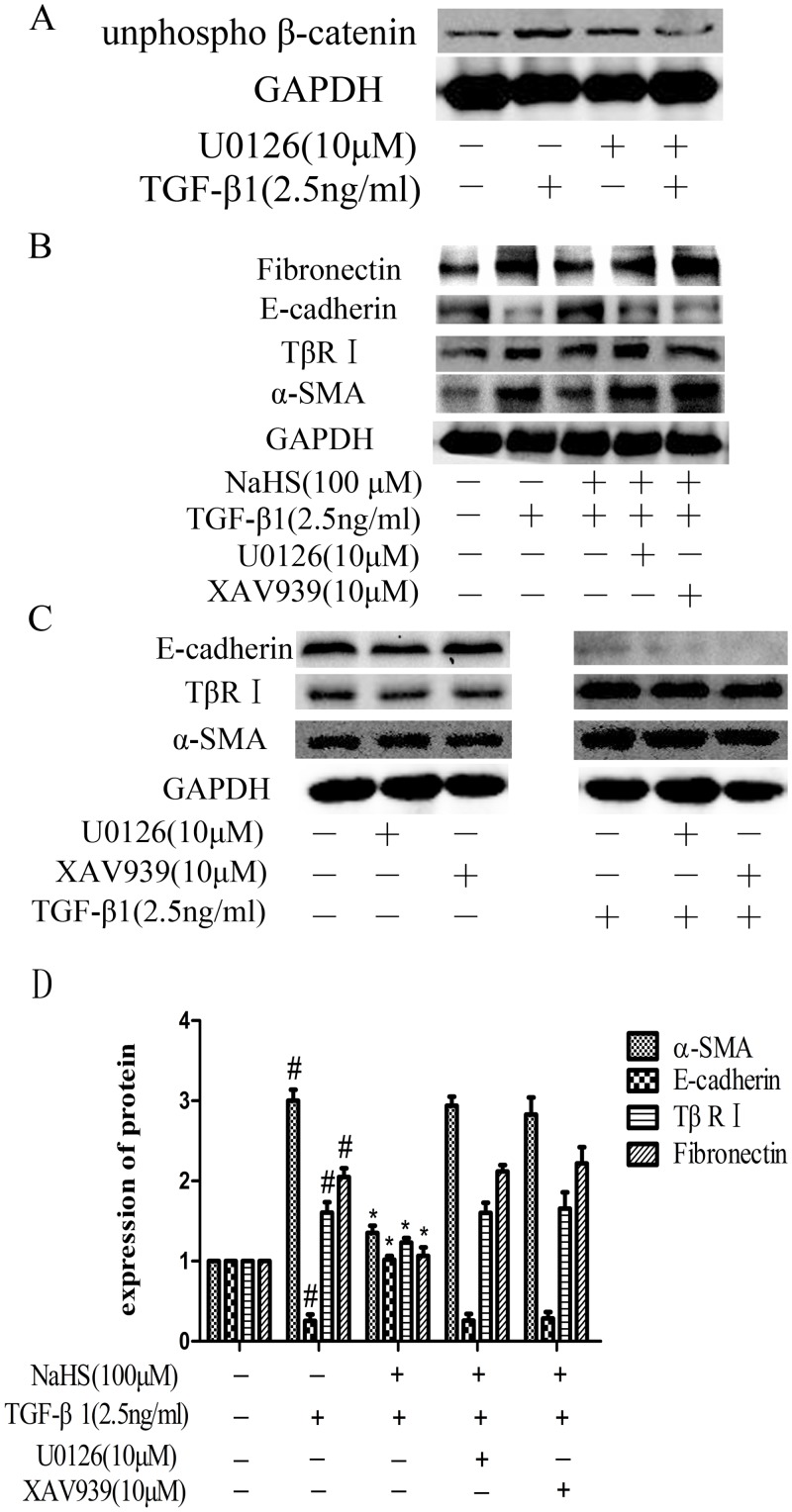
The anti-EMT effect of NaHS on HK-2 cells was blocked by the inhibitors of ERK1/2 and β-catenin. (A) The expression of non-phosphorylated β-catenin under the condition of ERK signal was blocked. ERK1/2 inhibitor U0126 (10μM) was given to HK-2 cells for 1 hour and then cells were incubated with TGF-β1 for 36 hours. (B) Effects of NaHS (100μM) on the expression of fibronectin, E-cadherin,TβR I and α-SMA in the presence of TGF-β1 and U0126 or the β-catenin inhibitors XAV-939. Pretreatment by NaHS (100μM) is given to HK-2 cells for 12 hours and then U0126 (10μM) or XAV-939 (10μM) were given to the cells for 1 hour before they were incubated with TGF-β1 for the following 36 hours. (C) Graphical representation of the relative quantification for the protein level. The relative values were calculated by the density of targeted proteins vs GAPDH (%). The values of mean ± SEM (n = 3) were gained from relative abundance quantified by densitometry and normalized to GAPDH. ^#^P<0.05 vs. control group, *P<0.05 vs TGF-β1 group. One-way ANOVA followed by Tukey’s multiple comparison test.

As shown above, NaHS affected the activation of ERK1/2 and β-catenin (Figs 4, 5 and 6). We further explored the importance of ERK1/2 and β-catenin in the anti-EMT effect of NaHS, by blocking them with their respective inhibitors. We excluded the influence of U0126 and XAV939 themselves on the normal growth and establishment of EMT model in HK-2 cells (Fig 7C). When blocking ERK1/2 using U0126, the pharmacological inhibitor of ERK1/2, the expression level of non-phosphorylated β-catenin was decreased even in the presence of TGF-β1 (Fig 7A). This resulted loss of β-catenin activation, suggests crosstalk between ERK1/2 and β-catenin. We then blocked both ERK1/2 and β-catenin signaling pathways using U0126 and XAV939, the pharmacological inhibitor of β-catenin, and then detected whether NaHS could still attenuate EMT. As shown in Fig 7, in the presence of U0126 or XAV939, NaHS (100μM) failed to decrease the expression of fibronectin, α-SMA and TβR I or increase the expression of E-cadherin in HK-2 cells compared to the group treated with NaHS (100μM) without the inhibitors (Fig 7B & 7D).

### 8. NaHS attenuates the TGF-β1-induced EMT through Wnt/catenin pathway

**Fig 8:**
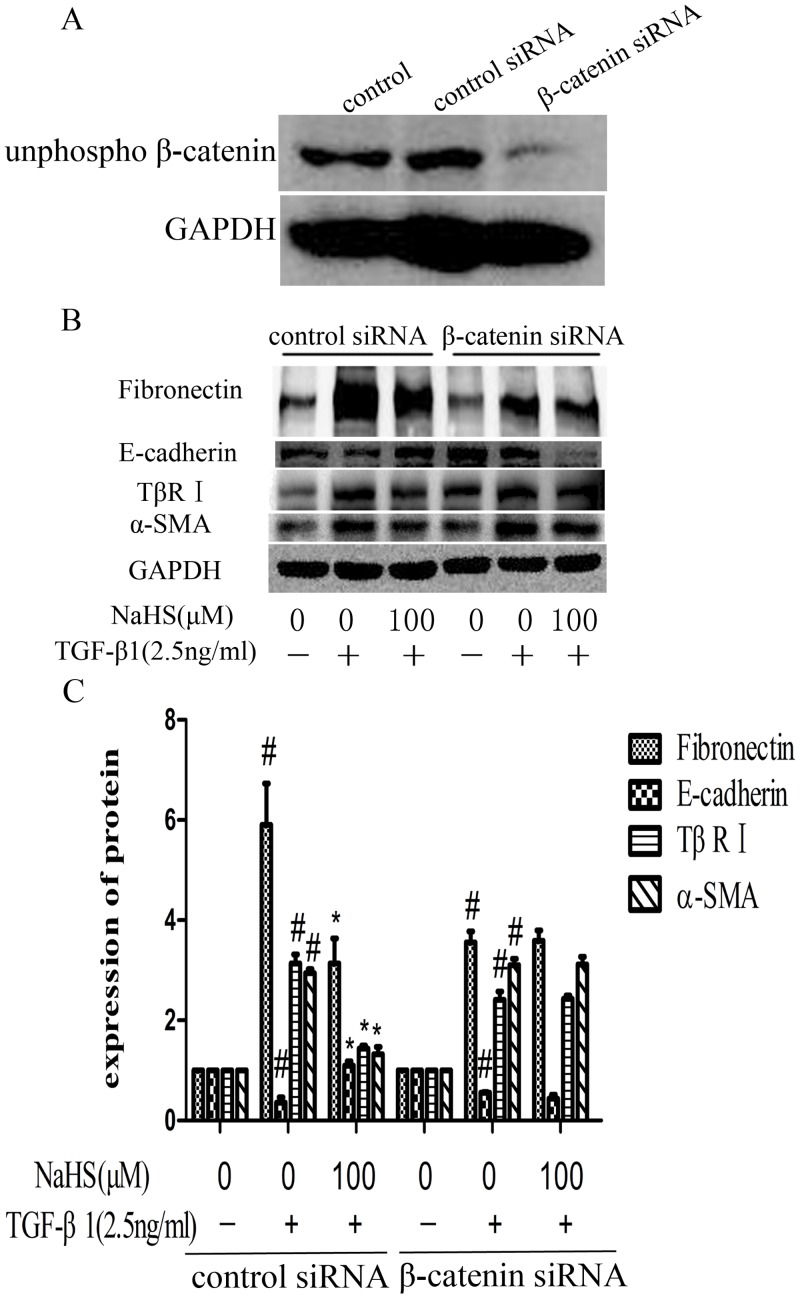
NaHS attenuates the TGF-β1 induced EMT via Wnt/catenin pathway. (A) β-catenin knockdown efficiency by siRNA in HK-2 cells. The cells were transfected with β-catenin siRNA or control siRNA for 6 hours and the culture medium was replaced by fresh medium. Western blot assay was used to detect the expression level of non-phosphorylated β-catenin. (B) After transfected with β-catenin siRNA or control siRNA for 6 hours, HK-2 cells were incubated with NaHS (100μM) for 12 hours and then with TGF-β1 for the following 36 hours. The expression level of fibronectin, E-cadherin, TβR I and α-SMA were examined by western blot assay. (C) Graphical representation of the relative quantification for the protein level. The relative values were calculated by the density of targeted proteins vs GAPDH (%). The values of mean ± SEM (n = 3) were gained from relative abundance quantified by densitometry and normalized to GAPDH. ^#^P<0.05 vs. control group, *P<0.05 vs TGF-β1 group. One-way ANOVA followed by Tukey’s multiple comparison test.

In addition, we knocked down the expression of β-catenin at protein level by transfecting β-catenin siRNA into HK-2 cells. Negative siRNA was used as control. Western blotting confirmed the β-catenin knock down (Fig 8A). With β-catenin significantly being knocking down, NaHS (100μM) failed to decrease the expression of fibronectin, α-SMA and TβR I or increase the expression of E-cadherin, compared to negative siRNA group (Fig 8B & 8C). This result indicates that Wnt/catenin pathway may be the mechanism by which NaHS attenuates TGF-β1 induced EMT.

### 9. NaHS inhibits the migration of HK-2 cells induced by TGF-β1

**Fig 9:**
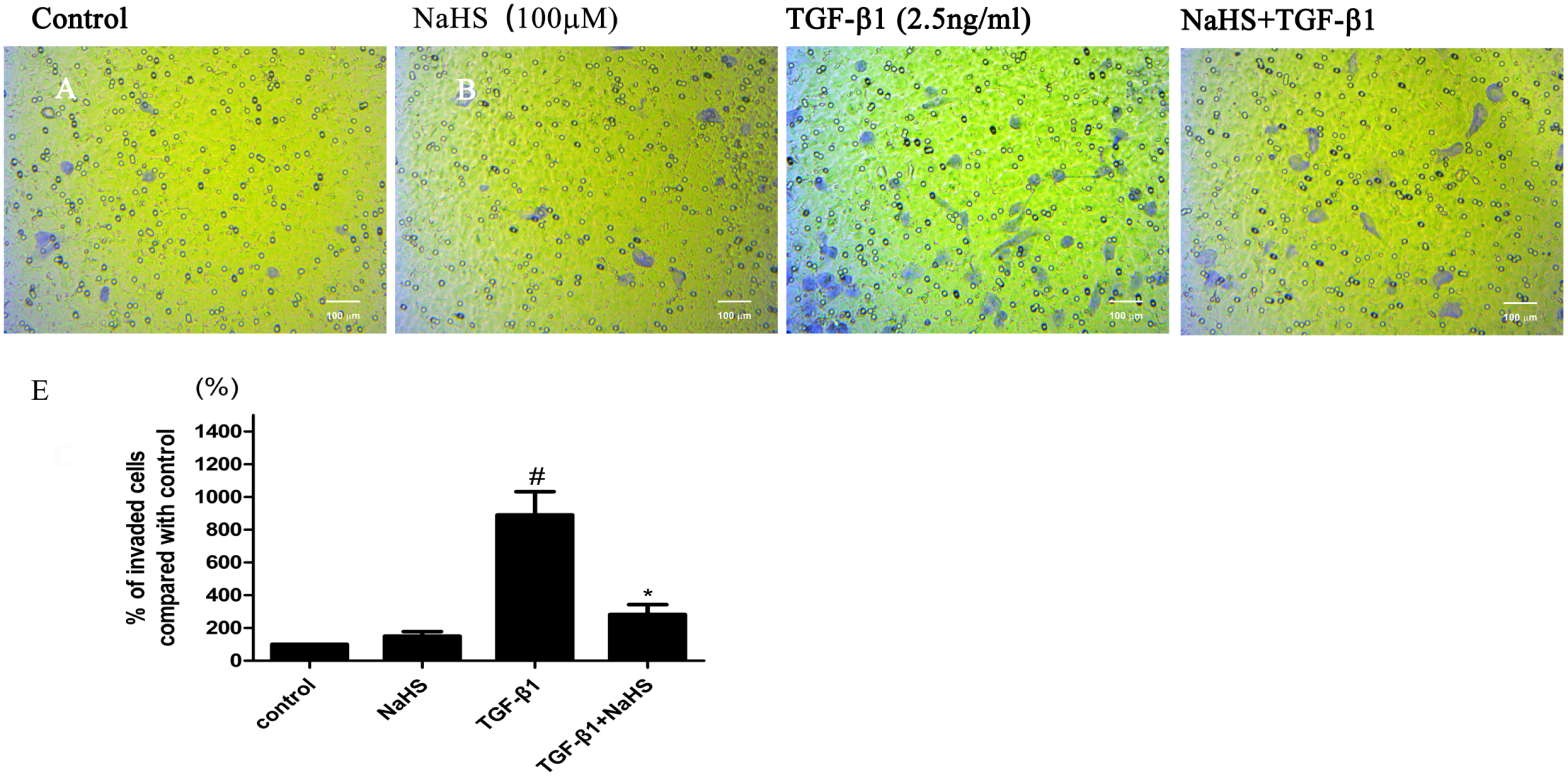
NaHS inhibits the migration of HK-2 cells induced by TGF-β1. (A,B,C,D) HK-2 cells were pretreated with or without NaHS (100μM) for 12 hours and then treated by TGF-β1 for 36 hours. Cells were detached and seeded to the upper section of the chamber, and then incubated for 24 h in a 5% CO2 incubator at 37°C. Purple color stands for cells that had gone through the membrane. (E) Graphical representation of the invaded cells. The values of mean ± SEM (n = 3) were gained from three randomly microscope fields in each chamber and normalized to control group. ^#^P<0.05 vs. control group, *P0.05 vs TGF-β1 group. One-way ANOVA followed by Tukey’s multiple comparison test.

We further evaluated the effects of NaHS on TGF-β1-induced mobility increase of HK-2 cells using transwell migration assay. As shown in Fig 9, TGF-β1 increased the mobility and invasive capacity of HK-2 cells. Treatment with NaHS significantly reduced the number of invaded cells and inhibited the migration of HK-2 cells (Fig 9).

## Discussion

Study by K-J Jung et. al. had linked H_2_S to TGF-β signaling in unilateral UO-induced kidney fibrosis in mice [4]. However, the molecular events underlying the effect of H_2_S on EMT remain unknown. As a significant step towards fibrogenesis, EMT presents the morphological changes associated with downregulation of E-cadherin and upregulation of most common mesenchymal markers such as vimentin, fibronectin, α-smooth muscle actin (α-SMA) [16]. In our study, we find out that under the pretreatment by NaHS, overexpression of α-SMA and fibronectin stimulated by TGF-β1 was attenuated, meanwhile, the loss of E-cadherin induced by TGF-β1 was significantly reversed (Fig 1). This effect of H_2_S inhibiting the process of EMT, can also be seen in human breast cancer and alveolar epithelia cells as well [17, 18], thus establish the anti-fibrotic role of NaHS in renal tubular cell line.

H_2_S is synthesized endogenously in mammals from the sulfur-containing amino acid l-cysteine by the action of several distinct enzymes [1–3], though only CBS was detected in HK-2 cells in our study (Fig 2). The other enzymes were not detected by Western blotting probably due to their low concentrations in HK-2 cells or the tissue-specific enzymatic pathways for H_2_S production. Further study can be carried out to determine whether a particular enzyme is the dominant in the entire renal tissue.

To investigate the molecular mechanism underlying the inhibition effect of H_2_S towards EMT in renal tubular epithelial cells, we first detected the levels of TβR II and TβR I expression under the treatment of NaHS. Results showed that NaHS could reduce TGF-β1-induced overexpression of both TβR II and TβR I (Fig 3) in the traditional TGF-β/Smad signaling which is a pathway most important to and highly depended by EMT [5]. In contrast, L. Sun et al. have shown that NaHS selectively inhibited TβR II in myocardium [19], suggesting that the effect of NaHS on TGF-β signaling may be tissue-specific. Further down the pathway, the phosphorylation of Smad2 and Smad3 were shown to be significantly reduced by the pretreatment with NaHS in myocardial collagen remodeling in spontaneously hypertensive rats and in TGF-β1-stimulated cultured cardiac fibroblasts [19], and in unilateral UO animal model [20].

Apart from TGF-β /Smads pathway, TGF-β receptors can also activate non-Smad signal pathways, including ERK pathway and others. Many studies showed that ERK1/2 could be phosphorylated when EMT occured [21], while NaHS abolished the increased phosphorylation of mitogen-activated protein kinase [20]. In serum-starved HK-2 cells co-treated by NaHS and TGF-β1, we found that NaHS significantly attenuated the TGF-β stimulated overexpression of phospho-ERK1/2 in cell lysates (Fig 4), though it did not affect the levels of phospho-p38/p38 and phospho-JNK/JNK (data not shown). When cells were treated by U0126 together with NaHS, the anti-EMT effect of NaHS was abolished (Fig 7), suggesting that the anti-EMT effect of NaHS in HK-2 cells relies on ERK1/2 signaling.

Many studies have shown there may exists crosstalk between TGF-β/Smad and Wnt/β-catenin pathways by interactions between intracellular proteins of the two pathways [22]. When β-catenin disassociate from the adherens junction complex, the interaction between Smad3 and Smad4, which are the intermediates in TGF-β signaling, and β-catenin is stimulated by TGF-β1 in the cytoplasm [13]. C.S. Kim et al. have reported that expression of β-catenin was increased in 4-hydroxy-2-nonenal and 4-hydroxy-2-hexenal induced EMT and established the relationship between Wnt/β-catenin signaling and renal tubular EMT [23]. B. Zhou et al. have shown that TGF-β1 induced convergence of β-catenin-dependent and canonical Smad3 signaling is critical during the development of TGF-β-induced EMT. And they found that ICG-001, a specific inhibitor of β-catenin/CBP interactions, prevented TGF-β1-induced EMT and α-SMA activation, demonstrating a novel requirement of CBP-dependent β-catenin signaling in the stimulation of the EMT target gene α-SMA by TGF-β [24]. In this study, we demonstrated, for the first time, that both total β-catenin and non-phosphorylated β-catenin expression levels were increased after being treated by TGF-β1 and the pretreatment with NaHS attenuated this change (Fig 5). Furthermore, NaHS reversed the nuclear translocation of non-phosphorylated β-catenin caused by TGF-β1 in HK-2 cells (Fig 6). In addition, when we blocked ERK1/2 signaling using U0126, the overexpression of the non-phosphorylated β-catenin stimulated by TGF-β1 perished, indicating that there exist a relationship between ERK and Wnt/catenin signaling pathway.

Generally speaking, our research gave evidence that hydrogen sulfide can inhibit the TGF-β1-induced EMT in renal tubular epithelial cells via both ERK-dependent and Wnt/catenin-dependent pathways. This may potentially indicate a new therapeutic approach to renal fibrosis.
